# Neurobehavioral, neurotransmitter and redox modifications in *Nauphoeta cinerea* under mixed heavy metal (silver and mercury) exposure

**DOI:** 10.1186/s13104-024-06852-2

**Published:** 2024-07-05

**Authors:** Olawande C. Olagoke, Opeyemi B. Ogunsuyi, Famutimi E. Mayokun, João B.T. Rocha, Ganiyu Oboh

**Affiliations:** 1 Division of Gastroenterology, Department of Medicine, Beth Israel Deaconess Medical Center, Harvard Medical School, Boston, MA USA; 2 Division of Translational Research and Technology Innovation, Department of Medicine, Beth Israel Deaconess Medical Center, Harvard Medical School, Boston, MA USA; 3 Department of Physiology, Kampala International University, Ishaka-Bushenyi, Uganda; 4 Department of Biomedical Technology, Federal University of Technology, P.M.B. 704, Akure, Nigeria; 5 Drosophila Research Lab, Functional Foods and Nutraceuticals Unit, Federal University of Technology, P.M.B. 704, Akure, Nigeria; 6 Department of Biochemistry, Federal University of Technology, P.M.B. 704, Akure, Nigeria; 7 Programa de Pos-graduacao em Bioquimica Toxicologica, Departamento de Bioquímica e Biologia Molecular, Centro de Ciências Naturais e Exatas (CCNE), Universidade Federal de Santa Maria, Santa Maria, RS 97105-900 Brazil; 8 Departamento de Bioquímica, Instituto de Ciências Básicas da Saúde, Universidade Federal do Rio Grande do Sul, Rua Ramiro Barcelos 2600-Anexo, Porto Alegre, RS 90035-003 Brazil

**Keywords:** Amalgam ban, Mercury toxicity, Silver toxicity, Neurotoxicity, 3Rs

## Abstract

Heavy metals are encountered in nature, and are used in several human endeavors, including in dental fillings. It is well known that the safety of metals depends on their chemical form, as well as the dose and route through which biological systems are exposed to them. Here, we used the *Nauphoeta cinerea* model to examine the mechanism by which salts of the heavy metals used in dental fillings – silver and mercury – exert their neurotoxicity. Nymphs exposed to heavy metals presented with reduced motor and exploratory abilities as they spent more time immobile, especially in the periphery of a novel object, and covered less distance compared with control nymphs. Exposure to AgNO_3_ and HgCl_2_ also exacerbated levels of oxidative stress markers (MDA & ROS) and the neurotransmitter regulators – AChE and MAO, while reducing antioxidant activity markers, both in biochemical (thiol & GST) and RT-qPCR (TRX, GST, SOD, Catalase) examinations, in neural tissues of the cockroach. The observed disruptions in neurolocomotor control, synaptic transmission and redox balance explain how heavy metal salts may predispose organisms to neurological disorders.

**Supplementary Information:**

The online version contains supplementary material available at 10.1186/s13104-024-06852-2.

## Introduction

Dental fillings with amalgams are considered safe, despite over a century of deliberations. However, a 2020 US FDA statement posited that certain pre-existing conditions could exacerbate the harmful effects of the mercury vapor that amalgams release [1], and the European Union (EU) parliament has voted to ban dental amalgam use and export from January 2025, while the British Dental Association (BDA) is more inclined toward a phasing-out amalgam fillings [2]. Indeed, continuous and chronic mercury vapor emission from dental amalgam is exacerbated during chewing, tooth brushing and hot fluid intake. Consequently, mercury vapor in absorbed into the lungs, saliva, dental pulp (via the dentinal tubules), as well as the brain and pituitary gland (via the nasal mucosa) [3, 4].

In the elemental state, the constituents of amalgam like silver and mercury are used in dentistry, meteorology, fashion, and finance, amongst other practical applications. On the other hand, organic mercury is the most toxic of the three forms of mercury, and exposure to heavy metal ions via ingestion, inhalation, and dermal contact damages cellular components (cell membrane, DNA, etc.), displaces essential metal ions from enzymes, deform and inactivate enzymes by interacting with amino acid residues on sulfur-containing enzymes, and destabilizes protein structure and function [5]. Subsequently, system-wide disorders, including neurological disorders ensue, and there are records of the development and progression of cancers [6, 7].

Given the increasing awareness that heavy metal toxicity depends on chemical form and concentration, and the understanding that biogeochemical cycling enables heavy metals to be converted from one form to the other in nature and in industrial settings [8, 9], we used the lobster cockroach to study the neurobehavioral, neurotransmitter and redox modifications that follow exposure to the salts of two major components of dental amalgam – silver and mercury. The cockroach has been extensively used to study the neurotoxic outcomes of exposure to organic mercury [10–14] and other xenobiotics [15–17], due to the similarity in neuronal signaling from insects to mammals [16, 18, 19]. We therefore explored using the alternate model to understand neurological effects of exposure to heavy metal salts (AgNO_3_ and HgCl_2_) in line with the need to replace, reduce, and refine (3Rs) the use of animals in biomedical research.

## Materials and methods

### Chemicals

Sigma Aldrich Co. (St Louis, Missouri, USA) supplied reduced glutathione and acetylthiocholine iodide. BDH Chemicals Ltd supplied acetic acid, potassium acetate and other chemicals. All chemicals were of analytical grade.

### *N. cinerea* husbandry and experimental protocol

*N. cinerea* was obtained at the CCNE, Universidade Federal de Santa Maria, Brasil. The cockroaches were maintained at the temperature and humidity of 24 ± 3 °C and 57–75%, respectively, with *ad libitum* access to water and feed as previously composed [16]. Size matched nymphs were randomly distributed into four groups of 20 nymphs each, including Control (basal diet), basal diet + 272 mg/g HgCl_2_, basal diet + 85 mg/g AgNO_3_, and basal diet + 272 mg/g HgCl_2_ + 85 mg/g AgNO_3_ and monitored for 7 days. The concentration and duration of exposure were based on the ratio of Hg: Ag in dental amalgam, and the result of pilot studies of feed intake (Table S1) and survival (Fig S1 & S2). Following the exposure period, neurolocomotor assessment was carried out, and nymph heads were excised while on ice, weighed, homogenized (100 mg head: 1 ml 0.1 M Phosphate buffer, pH 7.4 or 100 mg head: 1 ml TRIzol™) and centrifuged (2500 g x 10 min x 4 min) to produce supernatants for biochemical and PCR assays.

### Neurobehavioral, neurotransmitter and redox activity assessment

Neurolocomotor activity was filmed for 8 min in a novel environment (white plastic box; 19 × 12.5 × 5 cm) using a webcam mounted over the setup. Data from the video files were analyzed using the ANY-maze 6.0, Steolting, CO, USA video-tracking software [20].

Lipid peroxidation was estimated as described by Ohkawa et al. [21, 22]. 50 µL tissue homogenate was mixed with SDS (150 µL, 8.1%), 20% acetc acid in hydrogen chloride (250 µL, p.H 3.4) and TBA (250 µL, 0.6%). Th tissue homogenate was replaced with distilled water for the blanks. The setup was incubated (1 h x 95 ^o^C) and the product was read at 532 nm. Results were presented as µmol/mg protein.

H_2_O_2_ was used as an estimate of ROS (reactive oxygen species) levels as described by Hayashi et al. [23, 24]. Tissue homogenate was incubated at 37 ^o^C in sodium acetate buffer (57 mM, pH 4.8) for 5 min, before the addition of n-n-diethyl-para-phenylenediamine (2.5 mg/Ml) and ferrous sulphate solution (1.8 µM). The absorbance of the resulting product was read at 505 nm and compared against a H2O2 standard calibration curve. Results were expressed as Unit/mg protein.

Total thiol content was estimated by mixing the tissue homogenate (20 µL) with 5,5’-dithiobis-(2-nitrobenzoic acid) (0.5 mM), and potassium phosphate buffer (85 mM, pH 7.4) [25, 26]. Blanks were created without DTNB, and all reaction volumes were left at room temperature for 30 min before the product was read at 412 nm. Results were presented as mmol/mg protein.

Habig and Jakoby’s method of using 1-chloro-2,4-dinitrobenzene (CDNB) as a substrate for estimating glutathione-S-transferase activity was utilized [27, 28]. 100 µL tissue homogenate was mixed with ethylenediaminetetraacetic acid (1 mM), chloro-2, 4-dinitrobenzene (0.80 mM), glutathione as substrate (3.20 mM) and potassium phosphate buffer (70 mM, pH 7.0). The setup was left at room temperature for 10 min and read at 340 nm. Results were expressed as unit/mg protein.

The method of Ellman et al. was used to estimate acetylcholinesterase activity [29, 30]. 30 µL tissue homogenate was mixed with phosphate buffer (10 mM, pH 7.4), 5,5-dithio-bis (2-nitrobenzoic) acid (1 mM), and acetylthiocholine iodide (0.8 mM). The product was read at 412 nm and results were presented as mmolAcSch/h/mg protein.

The method of Mcewen et al. was used to estimate monoamine oxidase content [31]. 50 µL tissue homogenate was mixed with potassium phosphate buffer (72 mM, pH 7.4), benzylamine (0. 5 mM), and distilled water (50 µl). The set up was incubated at 25 ^o^C for 30 min before the addition of 10% perchloric acid and centrifugation (1,500 g x 10 min) to get a clear product that was read at 280 nm. Results were presented as mmol/mg protein.

*Nauphoeta cinerea* primer sequence was designed for RT-qPCR analysis as earlier presented [16, 17]. Primer efficiency was determined from a 5-point pooled sample dilution, and 40 thermal cycles with a single cycle melt curve was used for the PCR. The Medtl™ System program of Gentier48R™ Real Time PCR with Photodiode detector system (Xi´an TianLong Science and Technology Co. Ltd, China) was used to access the results which were analysed with the 2^−ΔΔCT^ approach [17, 28, 32] and tubulin was used as the normalizer gene. The primer sequences used included: *TRX*: F – AGTATCCACGCGCCGTATT; R – TGGGGTCTGCTCCTTGTATC, *GST*: F – GGGACCTCTGAATGACGAAA; R – CATGCCGTCCAAATAATCAA, *SOD*: F – GTATTCTGGTGGCTGCGAAA; R – TAAACCCAACACAGAGCCTTG, *Catalase*: F – ACGAGATCCAGCATCTGACC; R – CTCCACGGTTATCCACAGGT, *Tubulin*: F – TTGCCAGTGATGAGTTGCTC. R – TAGTGGCTCCAGTGCAAGTC.

### Statistical analyses

Data were analysed via one-way Analysis of Variance (ANOVA) and Tukey’s multiple comparisons test and expressed as mean ± SD. Graph pad PRISM (V.8.0) was used for analyses and significance was set at *p* ≤ 0.05.

## Results

**Fig. 1:**
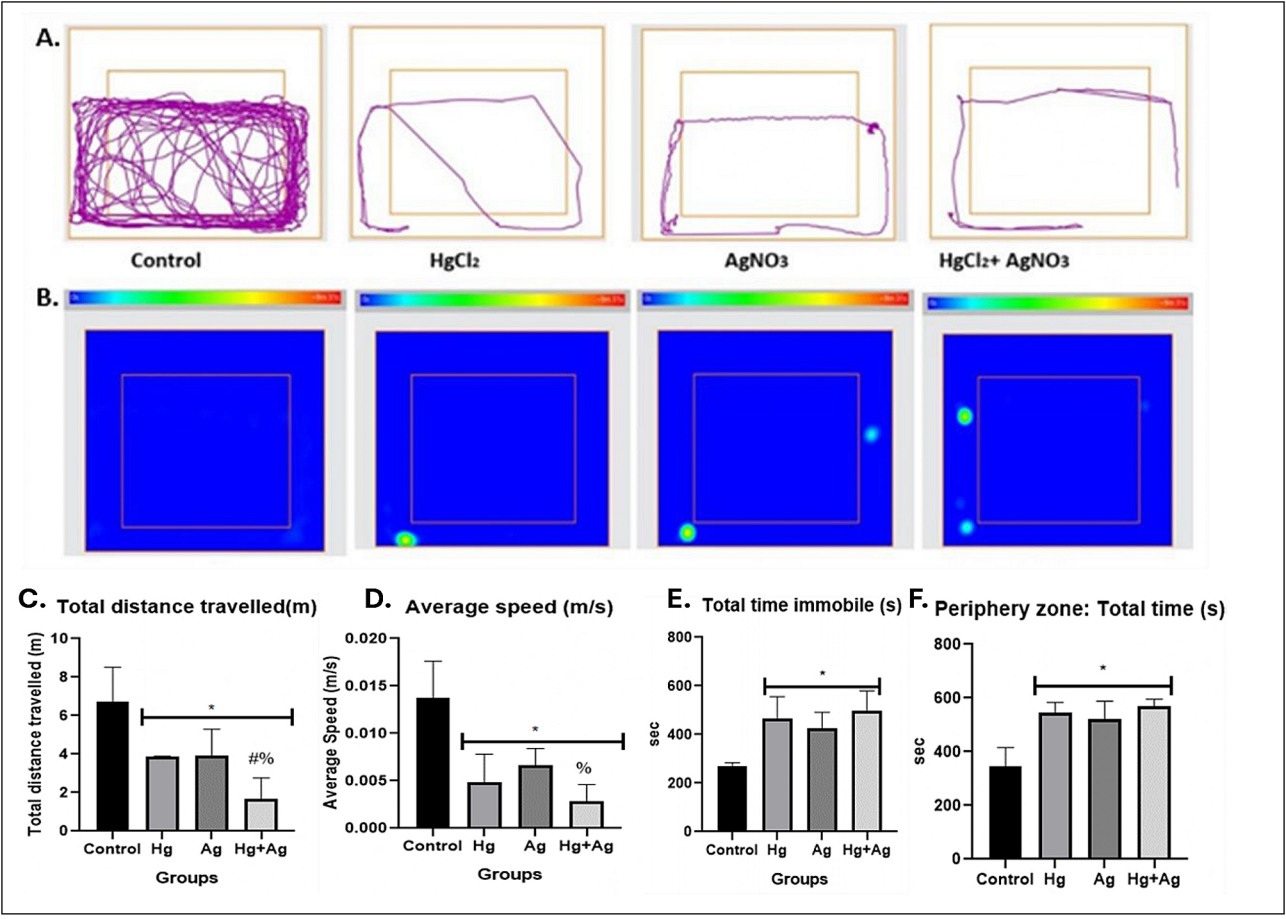
Exposure to mercury and silver salts disrupts motor and exploratory activities *in Nauphoeta cinerea*. The ANY-maze (Stoelting CO, USA) video tracking software was used to analyse recordings of an 8-minute trial that was observed in a novel environment. **A**. Track plot **B**. heat map **C**. Total distance travelled **D**. Average speed **E**. Total time immobile **F**. Total time in peripheral zone. Data shown as mean ± SD. * *p* < 0.05 against control, # *p* < 0.05 against HgCl_2_ only, % *p* < 0.05 against AgNO_3_ only

**Fig. 2:**
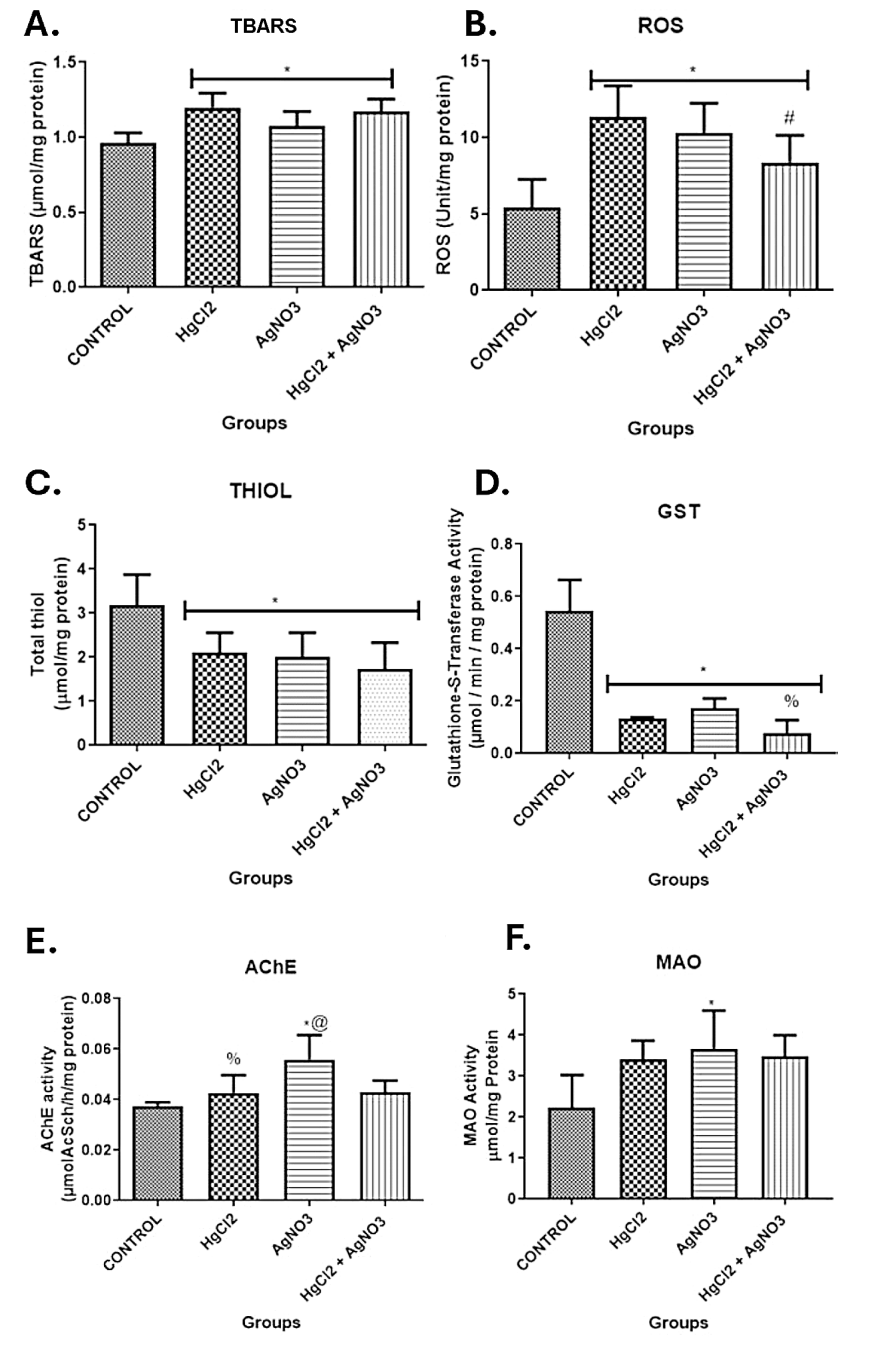
Effect of mercury and silver salts on oxidative stress (**A & B**), antioxidant activity markers (**C & D**) and neurotransmitter regulators (**E & F**) in neural tissues of *Nauphoeta cinerea.* Data shown as mean ± SD. * *p* < 0.05 against control, # *p* < 0.05 against HgCl_2_ only, % *p* < 0.05 against AgNO_3_ only

**Fig. 3:**
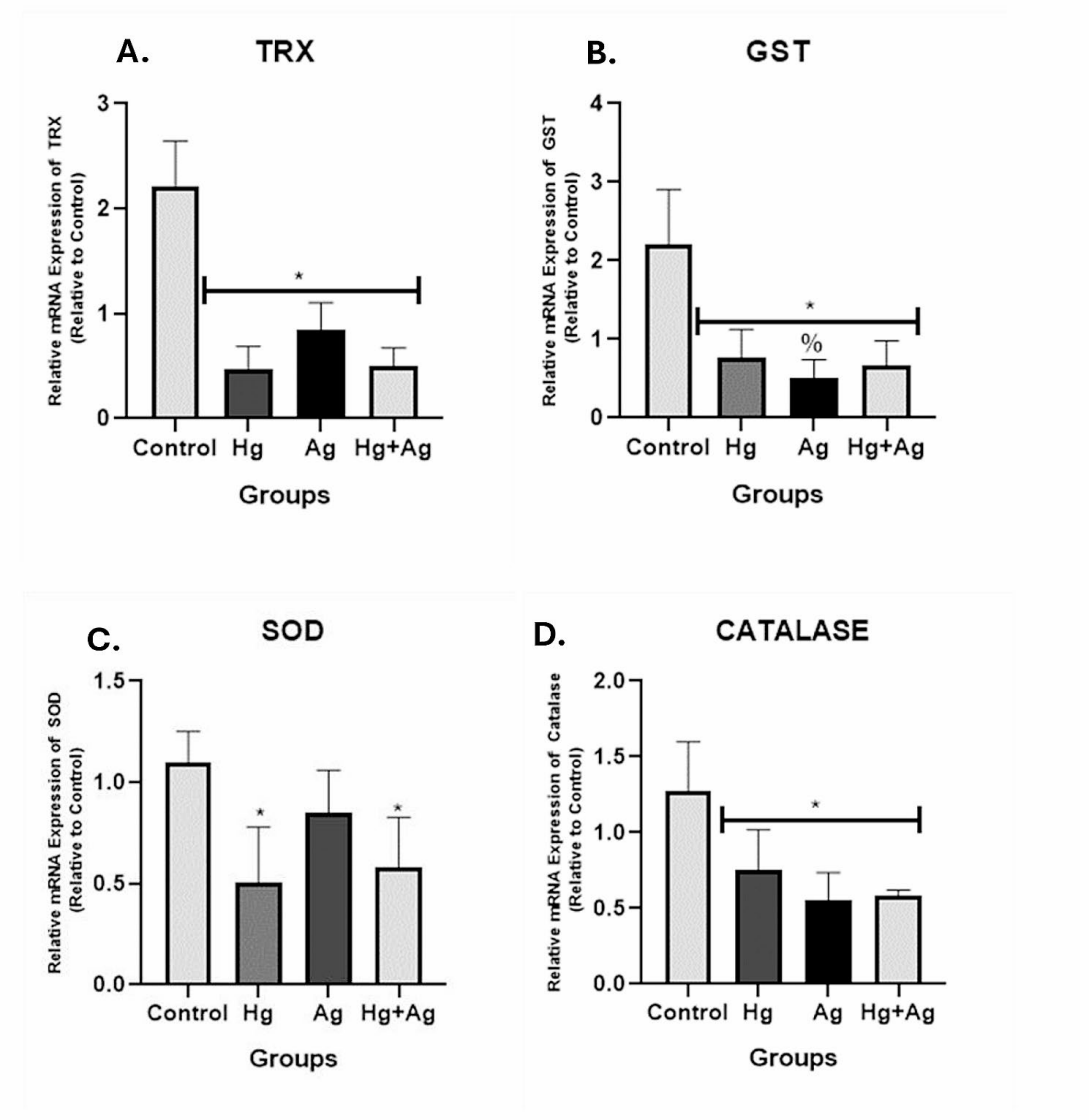
Effect of mercury and silver salts on gene expression of TRX, GST, SOD and catalase in neural tissues of *Nauphoeta cinerea.* Data shown as mean ± SD. * *p* < 0.05 against control, % p

Exposure to mercury and silver salts disrupted motor and exploratory activities in *Nauphoeta cinerea* as depicted by the track plots (Fig. 1A) and heat maps (Fig. 1B). The total distance travelled (Fig. 1C) and average speed (Fig. 1D) were also reduced, while the total time spent immobile (Fig. 1D) and the time spent in the periphery of the novel environment were increased (Fig. 1E) in the exposed nymphs. Heavy metal salts also increased oxidative stress markers (TBARS: Fig. 2A, ROS: Fig. 2B), reduced antioxidant activity (total thiol: Fig. 2C, GST: Fig. 2D), and increased AChE (Fig. 2E) and MAO (Fig. 2F) activity in the exposed nymphs. The mRNA levels of TRX (Fig. 3A), GST (Fig. 3B), SOD (Fig. 3C) and Catalase (Fig. 3D) were also reduced in nymphs exposed to heavy metal salts.

## Discussion

Heavy metals are ubiquitously present in the environment because of their diverse applications in day-to-day life [33], hence, they are closely monitored to understand their effect on humans, animals, and the environment. Genetic and environmental factors have been shown to determine the degree of heavy metal toxicity, and there are considerations for the dose and route of exposure. The cycling of heavy metals also makes it important to note the chemical form of the metal that an organism has been exposed to [34]. Here, we took advantage of the lobster cockroaches potential for modelling the reaction of chemicals with biological systems to study the salt forms of two heavy metals that are frequently used in dental fillings – silver and mercury.

Silver and mercury salts limited motor and exploratory activities in *N. cinerea* as the exposed nymphs spent more time immobile, especially in the periphery of the novel object, similar to previously reported heavy metal-induced neurolocomotor deficits [10, 35]. In the same vein, the neurological disorders that ensure from heavy metal exposure have been linked with raised levels of reactive oxygen species (ROS) with a consequent damage to cellular lipids, protein, and DNA [36]. The influence of heavy metals on ROS signaling differs with each chemical specie, but our report of increased oxidative stress markers and reduced antioxidant activity both via biochemical and RT-qPCR investigations in silver and mercury salts is similar to results shown during exposure to organic mercury [10–12, 37, 38].

Deranged synaptic transmission and neurotransmitter metabolism also contribute to heavy metal-induced neurological dysfunction [39]. Here, exposure to silver and mercury salts elevated AChE and MAO levels in the neural tissues of cockroaches, reminiscent of increased hippocampal activity of AChE and MAO which potentiates Aβ peptide formation in dementia [40–42].

## Conclusion and strength of the study

Silver and mercury salts (both individually and in combination) destabilize neuromotor control, synaptic transmission, and neural redox homeostasis, in a manner that might predispose to neurological disorders. This study aptly showcases *Nauphoeta cinerea*’s potential for modelling chemical reactions in biological systems, in line with the 3R’s approach to toxicity and safety assessment.

### Limitation and future perspectives

It is important to understand how individual metals affect biological systems, but exposure to heavy metals in nature is often in several chemical forms and combinations. Hence, new approach methodologies (NAMs) would be required to extrapolate toxicity assessment for the exposure of humans, animals and the environment to xenobiotics.

### Electronic supplementary material

Below is the link to the electronic supplementary material.

Supplementary Material 1

## Data Availability

Data is provided within the manuscript and supplementary material. Further information is available from O.C.O upon reasonable request.

## Abbreviations

3Rs: Replacement, reduction and refinement
US FDA: United States Food and Drug Administration
EU: European Union
RT-qPCR: Quantitative reverse transcription polymerase chain reaction
AgNO3: Silver nitrate
HgCl2: Mercury (II) chloride
MDA: Malondialdehyde
ROS: Reactive oxygen species
AChE: Acetylcholinesterase
MAO: Monoamine oxidase
TRX: Thioredoxin
NAMs: New Approach Methodologies
