# Supplementary material for: Neurobehavioral, neurotransmitter and redox modifications in Nauphoeta cinerea under mixed heavy metal (silver and mercury) exposure
Source: BMC Res Notes. 2024 Jul 5;17:188. doi: 10.1186/s13104-024-06852-2 (PMC11227235; doi:10.1186/s13104-024-06852-2)
Supplement: Supplementary file 1 — Supplementary Material 1 [file 13104_2024_6852_MOESM1_ESM.docx]

**Supplementary Data for: Neurobehavioral, neurotransmitter and redox modifications in *Nauphoeta cinerea* under mixed heavy metal (silver and mercury) exposure.**

Olawande C. Olagoke^1,2,3^, Opeyemi B. Ogunsuyi^4,5,7^, Famutimi E. Mayokun^4,5^, João B.T. Rocha^7,8^, Ganiyu Oboh^5,6^

^1^ Division of Gastroenterology, Department of Medicine, Beth Israel Deaconess Medical Center, Harvard Medical School, Boston, MA, USA

^2^ Division of Translational Research and Technology Innovation, Department of Medicine, Beth Israel Deaconess Medical Center, Harvard Medical School, Boston, MA, USA

^3^ Department of Physiology, Kampala International University, Ishaka-Bushenyi, Uganda

^4^ Department of Biomedical Technology, Federal University of Technology, P.M.B. 704, Akure, Nigeria

^5^ Drosophila Research Lab, Functional Foods and Nutraceuticals Unit, Federal University of Technology, P.M.B. 704, Akure, Nigeria

^6^ Department of Biochemistry, Federal University of Technology, P.M.B. 704, Akure, Nigeria

^7^Programa de Pos-graduacao em Bioquimica Toxicologica, Departamento de Bioquímica e Biologia Molecular, Centro de Ciências Naturais e Exatas (CCNE), Universidade Federal de Santa Maria, 97105-900, Santa Maria, RS, Brazil

^8^ Departamento de Bioquímica, Instituto de Ciências Básicas da Saúde, Universidade Federal do Rio Grande do Sul, Rua Ramiro Barcelos 2600-Anexo, 90035-003, Porto Alegre, RS, Brazil

*Corresponding author:

Olawande C. Olagoke; [olawande.olagoke@kiu.ac.ug](mailto:olawande.olagoke@kiu.ac.ug)

**Table S1. Average Feed Intake**

| **Experiment 1** | | **Experiment 2** | |
| --- | --- | --- | --- |
| **Group** | **Average Feed Intake (g)** | **Group** | **Average Feed Intake (g)** |
| Control | 0.89±0.31 | Control | 1.31±0.34 |
| 136 mg/g HgCl_2_ | 0.78±0.23 | 272 mg/g HgCl_2_ | 0.65±0.25 |
| 272 mg/g HgCL_2_ | 0.73±0.10 | 85 mg/g AgNO_3_ | 1.10±0.26 |
| 544 mg/g HgCl_2_ | 0.62±0.09 | 272 mg/g HgCl_2_ + 85 mg/g AgNO_3_ | 0.78±0.16 |
| 42.5 mg/g AgNO_3_ | 0.79±0.12 |  |  |
| 85.0 mg/g AgNO_3_ | 0.88±0.15 |  |  |
| 170 mg/g AgNO_3_ | 0.88±0.19 |  |  |

**Figure S1**: Kaplan–Meier survival analysis of cockroach survival after a 7-day dietary exposure to 136, 272, and 544 mg/g HgCl_2_ & 42.5, 85, and 170 mg/g AgNO_3_. Log-rank (Mantel-Cox) test revealed significant (*P* = 0.05) reduction in survival in the groups exposed to doses of mercury and silver salts, except 544 mg/g HgCl2, which corresponds to reduced feed consumption, and 42.5 mg/g AgNO_3_, which did not induce adverse effect in the cockroach.

**Figure S2**: Kaplan–Meier survival analysis of cockroach survival after a 7-day dietary exposure to 272 mg/g HgCl_2_, 85 mg/g AgNO_3_ and 272 mg/g HgCl_2_ + 85 mg/g AgNO_3_.
